# Research Progress of Ozone/Peroxymonosulfate Advanced Oxidation Technology for Degrading Antibiotics in Drinking Water and Wastewater Effluent: A Review

**DOI:** 10.3390/molecules29051170

**Published:** 2024-03-06

**Authors:** Hai Lu, Xinglin Chen, Qiao Cong, Qingpo Li, Xiaoyan Wang, Shuang Zhong, Huan Deng, Bojiao Yan

**Affiliations:** 1 Key Laboratory of Songliao Aquatic Environment, Ministry of Education, Jilin Jianzhu University, Changchun 130118, China; luhai@jlju.edu.cn (H.L.); chenxinglin@student.jlju.edu.cn (X.C.); congqiao@jlju.edu.cn (Q.C.); liqingpo@student.jlju.edu.cn (Q.L.); wangxiaoyan@student.jlju.edu.cn (X.W.); 2 Key Laboratory of Groundwater Resources and Environment, Ministry of Education, Jilin University, Changchun 130021, China; zhongshuang@jlu.edu.cn; 3 College of Visual Arts, Changchun Sci-Tech University, Changchun 130600, China; 100343@cstu.edu.cn

**Keywords:** antibiotics, O_3_/PMS, advanced oxidation process, hydroxyl radical, sulfate radical

## Abstract

Nowadays, antibiotics are widely used, increasing the risk of contamination of the water body and further threatening human health. The traditional water treatment process is less efficient in degrading antibiotics, and the advanced oxidation process (AOPs) is cleaner and more efficient than the traditional biochemical degradation process. The combined ozone/peroxymonosulfate (PMS) advanced oxidation process (O_3_/PMS) based on sulfate radical (SO_4_^•−^) and hydroxyl radical (•OH) has developed rapidly in recent years. The O_3_/PMS process has become one of the most effective ways to treat antibiotic wastewater. The reaction mechanism of O_3_/PMS was reviewed in this paper, and the research and application progress of the O_3_/PMS process in the degradation of antibiotics in drinking water and wastewater effluent were evaluated. The operation characteristics and current application range of the process were summarized, which has a certain reference value for further research on O_3_/PMS process.

## 1. Introduction

As an emerging pollutant, pharmaceuticals and personal care products (PPCPs) include various antibiotics and other common drugs [1,2,3,4]. These substances enter the environment through various channels, including veterinary medicine, agricultural medicine, human medicine, and the use of cosmetics. Antibiotics are small molecular substances synthesized by secondary metabolism after organisms grow to a certain stage, and their function is to produce resistance to pathogenic microorganisms. Since the discovery of penicillin, antibiotics have been widely used to prevent or treat infectious diseases in human and animal bodies. According to the data [5,6], between 2000 and 2015, the global use of antibiotics increased by 65%. In recent years, the use of antibiotics has increased due to the prevalence of COVID-19. According to the report [7,8], antibiotics are used globally in an amount of about 200,000 tons/year, among which, China has the highest consumption and output of antibiotics in the world. Antibiotics result in toxic effects of refractory parts on aquatic organisms in the water environment, and the residual antibiotics in the water promote the formation of drug-resistant bacteria. Antibiotic microbial resistance (AMR) is carried by antibiotic resistant bacteria (ARB) and expressed by antibiotic resistant genes (ARGs). Therefore, the accumulation of antibiotics in the water body results in the production of ARB and ARGs [9,10].

Research has shown that with the rapid development of the pharmaceutical industry and animal husbandry, more and more antibiotics are being used every year as veterinary drugs and feed in many countries across the globe. Nearly 90% of antibiotics enter various water bodies through parent or metabolites and cannot be absorbed by human beings and animals [11]. The detection of antibiotics in surface water body, drinking water, and sewage effluent has been reported widely, with a concentration ranging from ng/L–μg/L [12,13,14]. Although there are few residues, the long-term persistence of antibiotics affects the stability and health of the environment and organisms. Therefore, an economical and effective treatment method for antibiotic wastewater becomes indispensable.

In the process of antibiotic degradation, AOPs can produce the two highly active free radicals of hydroxyl radical (•OH) and sulfate radical (SO_4_^•−^) to oxidize the target pollutants [15]. Both •OH and SO_4_^•−^ have strong oxidizability, which means they can react with most organic matter and remove various antibiotic pollutants efficiently. Therefore, the process shows good prospects for application in the treatment of antibiotic wastewater and is expected to replace the traditional process. For example, O_3_/PMS oxidation, Fenton/Fenton-like oxidation, photocatalytic oxidation, electrochemical oxidation, and O_3_/H_2_O_2_ oxidation are recognized as effective treatment processes for removing organic pollutants from wastewater. In recent years, perovskite-based oxides have shown high catalytic efficiency in AOPs, in which non-free radical and free radical optimization pathways may occur simultaneously. Xu X. et al. [16] reviewed the latest progress of double perovskite in various catalytic reactions such as electrocatalysis and photocatalysis. Yang L. et al. [17] uses Ruddlesden–Popper (RP) layered perovskite oxide as the material to activate PMS. The active oxygen in RP perovskite has high activity and mobility and promotes the formation of non-radical singlet oxygen. Through the adjustment and optimization of singlet oxygen, LaSrCo_0.8_Fe_0.2_O_4,_ which can effectively activate PMS, was found.

Table 1 listed several currently prevailing AOPs, some of which are combined to further improve the degradation efficiency. Compared with other AOPs, the O_3_/PMS process can produce SO_4_^•−^ with higher selectivity and longer half-life. The O_3_/PMS has broad application prospects in the field of antibiotics, in the treatment of drinking water and wastewater effluent [18].

Because the O_3_/PMS process has the advantages of being green, highly efficient, and brings a strong oxidation ability, and at the same time residual antibiotics in water environments are gradually increasing, the development of the O_3_/PMS process and the degradation of antibiotics have attracted more and more attention from scholars. In this review, the reaction mechanism of the O_3_/PMS advanced oxidation process, the latest progress in treating antibiotic pollutants in water based on this process, and the countermeasures for by-products generated by the O_3_/PMS system were described in detail. Compared with other reviews, firstly, this review focused on the treatment of antibiotic pollutants, and specifically summarizes the efficiency of AOPs based on O_3_/PMS to degrade antibiotic pollutants in drinking water and wastewater effluent. Therefore, it is convenient for scholars who have just stepped into the field of AOPs to read. Secondly, most of the objects reviewed in this paper are representative antibiotic pollutants with high concentration in water. Finally, the by-products produced in the process of pollutant degradation and the corresponding inhibition methods were summarized. Therefore, this paper provided reference for further study of the O_3_/PMS process and offered both theoretical support and a scientific basis for the development of antibiotic degradation process in the water body.

## 2. Study on the Application of O_3_/PMS Advanced Oxidation Technology in the Treatment of Water Body Containing Antibiotics

### 2.1. Proposal of O_3_/PMS Advanced Oxidation Technology

Ozone oxidation is mainly used in the field of water and wastewater treatment, including drug source treatment in medical wastewater, post treatment of secondary sewage effluent, etc. [24,25,26]. While the combined process of ozone oxidation with other oxidants/catalysts generally exhibits a high degradation effect in treating antibiotic wastewater, the experiments showed that [27] ozone is effective in disinfection, decoloration and removal of micro-pollutants. Ozone is a strong oxidant with the main property of generating •OH in water. Ozone itself is a selective strong oxidant, the order of oxidation being as follows: alkene > amine > phenol > polycyclic aromatic hydrocarbon > alcohol > aldehyde > alkane. Ozone is likely to attack the double bonds of organic compounds, activated aromatic groups, and unprotonated amines [28]. Its reaction rate is much faster than that of ozone molecules, and its reaction rate constant is 10^8^–10^10^ mol^−1^·s^−1^ [29,30,31]. Therefore, the degradation of micro-pollutants by O_3_ is usually achieved through the co-action of molecular O_3_ and •OH. However, for the refractory pollutants in water, the oxidation efficiency of O_3_ alone is low, because only a small amount of •OH is produced by O_3_ decomposition and the molecular O_3_ is selective.

Therefore, •OH-based AOPs have attracted wide attention. The combined process of O_3_ and hydrogen peroxide (H_2_O_2_) is one of the most common advanced oxidation technologies used to degrade pollutants by •OH [32]. Table 2 showed the reaction equation for the O_3_/H_2_O_2_ system, see reference for detailed principles [33].

**Figure 1:**
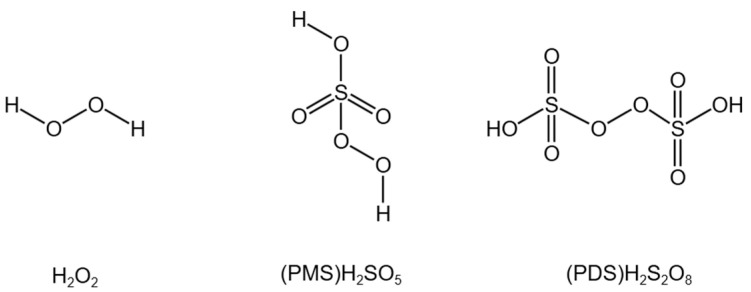
Molecular formulas of H_2_O_2_, PMS and PDS.

As a strong oxidant, SO_4_^•−^ has a higher oxidation–reduction potential (E_0_ = 2.5–3.1 V) and higher selectivity than •OH. In addition to this, the reaction of SO_4_^•−^ with micro-pollutants is less affected by alkalinity and natural organic matter [34,35,36]. SO_4_^•−^ is formed by the activation of persulfate (PS) (that is, persulfacte (PDS) and peroxymonosulfate (PMS)). Both PMS and PDS have strong oxidizability, but when PMS and PDS are used alone to degrade organic matter, the oxidation effect is not obvious. Therefore, a certain activation mode is required to activate them to produce SO_4_^•−^ and •OH with strong oxidizability. Activation methods mainly include ultraviolet irradiation, heating or addition of transition metals and carbon materials, etc. PS has the same peroxy bonds as H_2_O_2_, generating free radicals in the form of breaking peroxy bonds, the only difference being that they form SO_4_^•−^ and •OH, respectively [37]. Cong J. et al. [38] proposed that the O_3_-activated PMS process could enhance the degradation effect of chlorobenzoic acid (*p*CBA), and verify that PMS had the effect of promoting the generation of free radicals, similar to H_2_O_2_. In addition, the research of Li S. et al. [39] proved that the high chemical reactivity and low kinetic stability of PMS promoted it to react with O_3_. The molecular formulas of H_2_O_2_, PMS and PDS are shown in Figure 1. PDS exists in a symmetrical structure, and the peroxy group is stable and can hardly react with O_3_ [30]. Therefore, extensive research has been conducted on the use of O_3_ to activate PMS, thereby simultaneously producing •OH and SO_4_^•−^, which can rapidly and effectively degrade a variety of micro-pollutants [33].

### 2.2. Reaction Mechanism of O_3_/PMS Advanced Oxidation Technology

**Figure 2:**
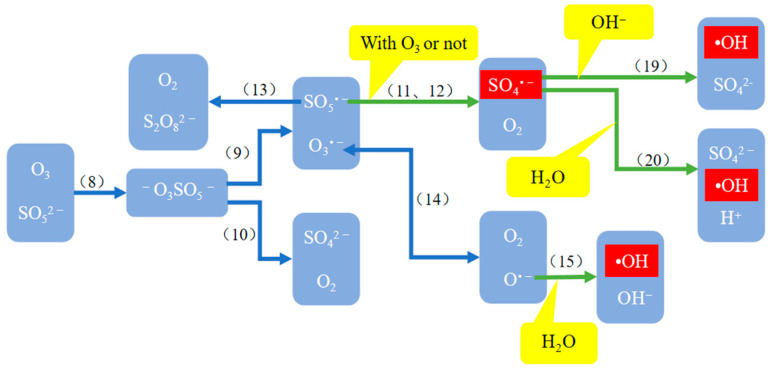
Reaction paths of •OH and SO_4_^•−^ in the O_3_/PMS system.

**Figure 3:**
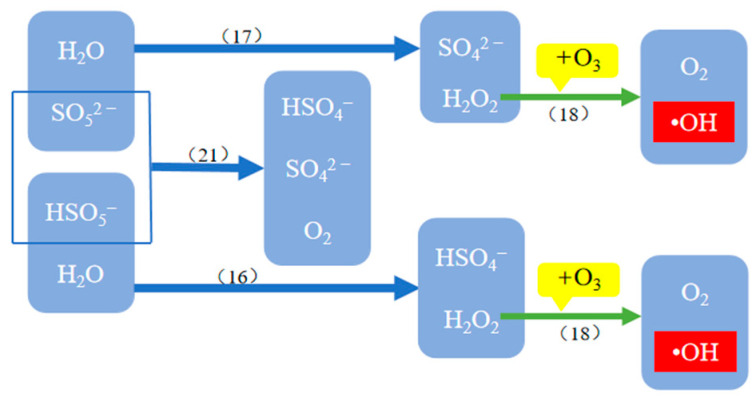
Reaction mechanism promoting the generation of •OH in the O_3_/PMS system.

Researchers have proposed various methods for activating PMS to generate SO_4_^•−^, including thermal decomposition, ultrasonic method, ultraviolet irradiation, alkali activation, laser flash method, transition metal or metal oxide catalysis, and so on [29]. Table 3 shows the reaction equations for the generation of •OH and SO_4_^•−^ in the O_3_/PMS system, see reference for detailed principles [20,33]. Figure 2 and Figure 3 show the reaction paths of •OH and SO_4_^•−^ in the O_3_/PMS system and the generation mechanism of •OH promoted by PMS and SO_4_^•−^, respectively (the serial numbers in the figures correspond to the serial numbers of reaction equations in Table 3).

### 2.3. Research Status of O_3_/PMS Advanced Oxidation Technology for Treatment of Antibiotics in Drinking Water and Wastewater Effluent

#### 2.3.1. Treatment Efficacy of O_3_/PMS Advanced Oxidation Technology for Antibiotics in Drinking Water and Wastewater Effluent

Due to the incomplete degradation of pollutants in water by traditional water treatment methods, clean and efficient advanced oxidation technology has attracted extensive attention from scholars. The O_3_/PMS process has more advantages in oxidation and can be used for the treatment of antibiotic pollutants in the water body.

**Figure 4:**
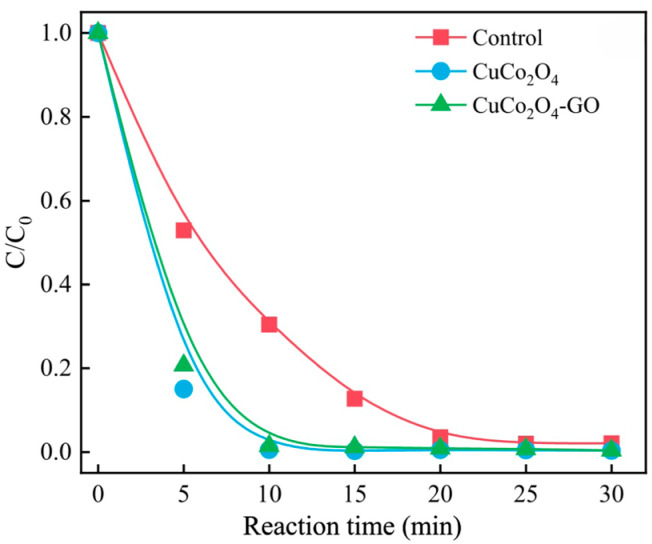
The removal effect of different reaction systems on SMX [43]. Reproduced with permission from Ref. [43]. Copyright 2022, Elsevier.

Sulfame-thoxazo (SMX), sulfamethazine (SMZ), and sulfadiazine (SDZ) are typical sulfonamide antibiotics in drinking water and wastewater effluent. Research has shown that the sulfate radical-based advanced oxidation process (SR-AOPs) can effectively degrade SMX, such as Fe^0^/PS [40], Fe(II)/PS [41], Co(II)/PMS [42]. Liu Z. et al. [43] studied the degradation of SMX by adding novel catalysts CuCo_2_O_4_-GO and CuCo_2_O_4_ as heterogeneous catalysts in the O_3_/PMS process. Liu Z. et al. prepared CuCo_2_O_4_ and CuCo_2_O_4_-GO nanoparticles by sol-gel method and hydrothermal method, respectively, and synthesized CuCo_2_O_4_-GO with different proportions of GO (5%, 10%, 20%, and 40%). The experiment was carried out in a 1000 mL hemispherical glass bottle. The experimental conditions were as follows: the temperature was 20 ± 2 °C, the pH was 7.0, the initial concentration of SMX was 30 μmol/L, the concentration of PMS was 100 μmol/L, the concentration of O_3_ was 1.30 mg/min, and the concentration of CuCo_2_O_4_ and CuCo_2_O_4_-GO was 100 mg/L. The degradation rate of SMX reached more than 98% after 10 min in the O_3_/PMS/CuCo_2_O_4_ and O_3_/PMS/CuCo_2_O_4_-GO system, while the O_3_/PMS process could almost completely degrade SMX after 25 min. Figure 4 shows that the addition of CuCo_2_O_4_ and CuCo_2_O_4_-GO promoted the degradation of SMX and greatly shortened the time for complete degradation of SMX. It is an effective way to strengthen the degradation of SMX. Du X. et al. [44] adopted a PMS/(oxidation/coagulation) coupled ceramic membrane process to remove Fe^2+^, Mn^2+^, and SMZ. The experimental conditions were as follows: the initial concentration of SMZ was 400 μg/L, the concentration of PMS was 100 μmol/L, the concentration of O_2_ was 0.14 mg/L, the concentration of Fe(II) was 4.2 mg/L, the concentration of Mn was 1.0 mg/L, and the initial pH was 7.3. A large number of humus substances are oxidized by the •OH radical and SO_4_^•−^ radicals. The results showed that Fe^2+^ and Mn^2+^ in groundwater played an in situ catalytic role in the system, which accelerated the yield of •OH and SO_4_^•−^ and further oxidized SMZ or natural organic matter (NOM). The particularity of the method was that Fe^2+^ in the groundwater could be used to activate the PMS in situ. In the oxidation process, the NOM in the water was also degraded. In recent years, Lu H. et al. [45] studied the degradation of SDZ in water by the O_3_/PMS process and compared the experimental results with the O_3_ alone and the O_3_/UV method. The experiment was carried out in a cylindrical glass reactor with a volume of 6 L. The experimental conditions were as follows: the temperature was 25 ± 0.5 °C, the initial concentration of SDZ was 10 mg/L, the concentration of O_3_ was 3 mg/L, the concentration of PMS was 22.8 mg/L, the UV intensity was 0.9 mw/cm^2^, and the pH was 6.8 ± 0.1. In the O_3_/PMS system, the degradation rate of SDZ reached 95.9% at 12 min, and the degradation rate of SDZ was below the detection limit at 13 min. However, at 12 min, the degradation rates of O_3_ and O_3_/UV were only 73.8% and 76.5%, respectively. The time required for O_3_/PMS to degrade SDZ was about 7 min shorter than that required by the O_3_ and O_3_/UV processes. In the O_3_/PMS system, the most suitable pH range is weak alkalinity. O_3_/PMS could effectively degrade SDZ and fluorescent substances dissolved in water, which showed a good prospect for practical engineering applications.

Ciprofloxacin (CIP) is a quinolone antibiotic considered to be a high-risk environmental pollutant [46]. In recent years, Li S. et al. [47] proposed a pore confinement method based on green ball milling, in which Co-Ce nanoparticles were immobilized on a mesoporous silica support as a catalyst to promote the degradation of CIP using the O_3_/PMS process. The catalyst exhibited higher activity due to enhanced interfacial mediated electron transfer. The heterogeneous O_3_/PMS reaction was carried out in a cylindrical reactor containing 500 mL 10 ppm CIP solution. The experimental conditions were as follows: the initial concentration of CIP was 10 mg/L, the concentration of PMS was 0.4 g/L, the concentration of the catalyst was 0.2 g/L, and the initial pH was 2.8. The total organic carbon removal rate of CIP was more than 70% after the treatment by the O_3_/PMS/Co-Ce-CS process for 10 min. The catalyst provides new possibilities for the treatment of organic pollutants in water environment.

The isothiazolinone bactericide is an antibiotic insecticide [48]. Li A. et al. [49] studied the kinetics and mechanism of oxidative degradation of methyl-isothiazolinone (MIT) by ozone. The experiment was carried out in a reactor with a volume of 3 L. The research showed that ozone could react with MIT at a low temperature. The molar concentration of ozone should be more than four times higher than that of MIT to achieve a higher removal rate. Ozone could reduce the toxicity of MIT by converting sulfur atoms in MIT to sulfate ions. The primary ozonation products of MIT showed toxicity, but the toxicity could be minimized by the ozonation reaction for 80 min. Yang Z. et al. [50] used O_3_/PMS to degrade isothiazolinone bactericide. The batch degradation experiment was carried out in a 100 mL conical flask. The experimental conditions were as follows: phosphate buffered saline with a concentration of 5 mmol/L was used as the medium, the concentration of O_3_ was 42 μmol/L, the concentration of PMS was 21 μmol/L, the initial concentration of MIT and CMIT was 1 mg/L, and the pH was 7. O_3_/PMS could degrade MIT and Chloro-methyl-isothiazolin (CMIT) by 91% and 81.8%, respectively, within 90 s, which were 30.6% and 62.5% higher than those of ozone oxidation alone. The experimental results showed that the total generation amount of O_3_/PMS free radicals was 24.6 times higher than O_3_ alone, indicating that •OH and SO_4_^•−^ played a major role in the oxidation of MIT and CMIT. Compared with O_3_/H_2_O_2_, it was found that the reaction rate of the deprotonation component of O_3_/PMS was higher than that of O_3_/H_2_O_2_. This shows that PMS can consume ozone molecules efficiently and improve the utilization rate of ozone.

Both cefalexin (CFX) and Amoxicillin (AM) belong to β-lactam antibiotics. Nguyen V. et al. [51] developed 2-Co_2_SnO_4_ and 2-Co_2_SnO_4_@rGO nanocomposites with different compounding ratios using a sol-gel method. Then, these nanocomposites were used as heterogeneous catalysts to degrade CFX in the form of a O_3_/PMS/2-Co_2_SnO_4_@rGO system. The experiment was carried out in a cylindrical borosilicate glass reactor with a height of 450 mm and a diameter of 60 mm. The experimental conditions were as follows: the concentration of CFX was 100 mg/L, the concentration of catalyst was 0.3 g/L, the concentration of PMS was 300 mg/L, the O_3_ flow rate was 15 mL/min, and the initial pH was 7. The CFX degradation efficiencies of Co_2_SnO_4_@rGO/PMS and O_3_/Co_2_SnO_4_@rGO/PMS reached 95.07% and 99.07%, respectively, at the compounding ratio of 2-Co_2_SnO_4_ and 1-rGO. The synergistic effect of O_3_/Co_2_SnO_4_@rGO/PMS led to the production of more •OH and SO_4_^•−^, thereby increasing the degradation rate of CFX in the system. Therefore, it is feasible to adopt the nano-composite catalyst to activate PMS in combined ozone oxidation to degrade antibiotic residues in wastewater. Gholikandi G. et al. [52] degraded AM in wastewater from a sewage treatment plant using the O_3_/PMS process and compared it with O_3_/PS, O_3_/H_2_O_2_ and O_3_ alone processes. In this experiment, a cylindrical glass reactor with a diameter of 3 cm and a height of 40 cm was used. The experimental conditions were as follows: the temperature was 17.6 °C, the concentration of O_3_ was 0.083 mmol/L, the concentration of PMS was 0.09 mmol/L, the concentration of H_2_O_2_ was 1.05 mmol/L, the concentration of PS was 3 mmol/L, and the initial pH was 6.9 ± 0.1. The removal rate of AM by O_3_/PMS reached 90 ± 2% within 30 min, while that of O_3_/PS and O_3_/H_2_O_2_ methods was only about 60–70%. Therefore, the removal rate of AM by O_3_/PMS was higher than that of the other three methods, and it could be used as an effective new method for sewage disinfection and removal of organic pollutants.

In recent years, biochar has exhibited great potential in environmental remediation due to low cost, large specific surface area, high porosity, and high conductivity. Biochar-assisted methods have attracted more and more attention in the degradation of organic pollutants in water [53]. Fang G. et al. [54] carried out research on the utilization of manganese oxide/biochar composite (Mn_x_O_y_ @BC) to activate periodate (PI) to degrade oxytetracycline (OTC). The experimental conditions were as follows: the temperature was 25 °C, the concentration of PI was 0.25 mmol/L, the initial concentration of OTC was 20 mg/L, the concentration of Mn_x_O_y_ @BC was 0.3 g/L, and the pH was 3.0. The degradation rate of OTC in Mn_x_O_y_ @BC/PI system was almost 98%, and the TOC removal rate was 75%. Various characterization analyses also verified that the functional groups on the surface of biochar and manganese active species (Mn (II), Mn (III) and Mn (IV)) could effectively activate PI. The Mn_x_O_y_ @BC system has great room for improvement and great practical application potential. Miao J. et al. [55] improved the activation efficiency of persulfate heterogeneous catalysts by loading metals on N-doped carbon materials. They studied and synthesized a Co/NC composite catalyst derived from Co_3_O_4_/PPY and used it to activate PMS to degrade benzothiazole (BTH). The catalytic degradation experiment confirmed the synergistic catalytic effect of Co, C, and N on PMS activation. The experimental conditions were as follows: the concentration of Co/NC-0.4 was 0.05 g/L, the concentration of PMS was 0.4 g/L, the initial concentration of BTH was 20 ppm, and the pH was 7.21. BTH was completely degraded within 20 min at 25 °C. In addition, under the same conditions, the production rate of free radicals in the Co/NC-0.4/PMS system is faster than that in other systems, and the effective utilization rate of PMS is also improved. The metal leaching of Co/NC-0.4/PMS system is also obviously inhibited by the coating of NC layer.

The O_3_/PMS process is an effective technology for treating antibiotics in various water bodies. But the generation of by-products cannot be ignored in practical application, and appropriate measures should be taken to inhibit the generation of by-products.

#### 2.3.2. Inhibition of By-Product Generation in O_3_/PMS System by Adding Carbon Materials

**Figure 5:**
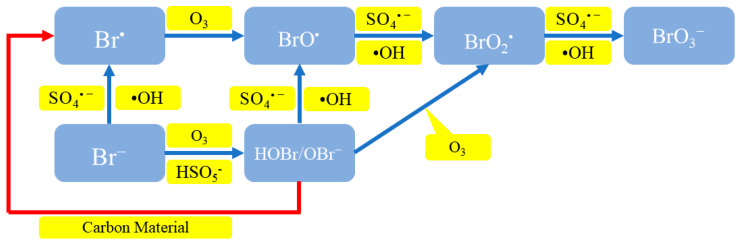
Formation mechanism of bromate in O_3_/PMS system (the red path represents the adding of carbon materials).

The O_3_/PMS process can effectively remove organic pollutants from water by utilizing •OH and SO_4_^•−^, and meanwhile, a large number of by-product bromate (BrO_3_^−^) is formed due to the existence of Br^−^ [56]. Figure 5 showed the formation mechanism of bromate in O_3_/PMS system [33].

Wen G. et al. [57] studied the formation mechanism of BrO_3_^−^ in the O_3_/PMS system. The first path was as follows: Br^−^ was oxidized to Br^•^ by SO_4_^•−^ and •OH, then Br^•^ reacted with O_3_ to form BrO_2_•, and then BrO• was oxidized to BrO_3_^−^ by SO_4_^•−^ and •OH; another path was that O_3_ oxidized Br^−^ to hypobromous acid (HOBr/OBr^−^), then HOBr/OBr^−^ reacted with O_3_ to form BrO_2_^•^, and BrO_2_^•^ continued to be oxidized to BrO_3_^−^. It could be seen that SO_4_^•−^ and •OH promoted the generation of by-products, so the O_3_/PMS process was more likely to produce BrO_3_^−^ than ozone oxidation alone.

**Figure 6:**
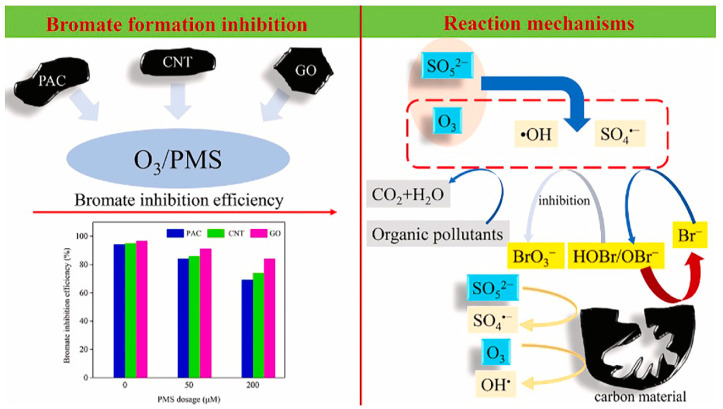
Effect of three materials on bromate formation efficiency in O_3_/PMS system [58]. Reproduced with permission from Ref. [58]. Copyright 2022, Elsevier.

Liu Z. et al. [43] proposed the addition of novel catalysts CuCo_2_O_4_-GO and CuCo_2_O_4_ as heterogeneous catalysts to the O_3_/PMS process for the degradation of SMX. Compared with CuCo_2_O_4_, CuCo_2_O_4_-GO not only retained the catalytic performance of CuCo_2_O_4_, but also inhibited the generation of BrO_3_^−^ in the O_3_/PMS system. HOBr/OBr^−^ was a decisive intermediate in the generation of BrO_3_^−^. The addition of CuCo_2_O_4_-GO mainly inhibited the further transformation of HOBr/OBr^−^ to reduce HOBr/OBr^−^ to Br^−^, thereby inhibiting the generation of BrO_3_^−^. The results showed that the optimal dosage of GO in CuCo_2_O_4_-GO was 20%. Under this ratio, CuCo_2_O_4_-GO had the highest first-order rate constant for the degradation of SMX and the lowest utilization efficiency of O_3。_This almost completely inhibited the generation of BrO_3_^−^, indicated that it promoted the decomposition of O_3_ to produce •OH, thereby reducing the reaction of O_3_ with Br^−^, and indirectly resulting in a large decrease in the generation of BrO_3_^−^. Meanwhile, when CuCo_2_O_4_-GO was added into actual water samples, the generation of BrO_3_^−^ was significantly inhibited. In addition, after CuCo_2_O_4_-GO was recycled three times, the degradation efficiency of SMX reached 90% within 10 min, and the inhibition efficiency of CuCo_2_O_4_-GO on BrO_3_^−^ could reach over 80% after the first two uses. Wen G. et al. [58] studied the addition of low-dose carbon materials to inhibit the generation of BrO_3_^−^ in the O_3_/PMS process. The added carbon materials included powdered activated carbon (PAC), carbon nanotubes (CNT) and graphene oxide (GO). The results showed that all three of the carbon materials played the role of reducing the intermediate product (HOBr/OBr^−^) in the reaction, thereby inhibiting the generation of BrO_3_^−^ in the system. The order of inhibition ability was as follows: GO > CNT > PAC. It can be seen from Figure 6 that adding low-dose carbon material is an effective way to promote the degradation of organic pollutants and control the formation of bromate. But, the inhibition methods are far more than that, including the adoption of NH_3_, Cl_2_-NH_3_, NH_3_-Cl_2_, and other treatment methods. See references [56,59] for details.

## 3. Conclusions

The O_3_/PMS process can simultaneously produce two highly active free radicals: •OH and SO_4_^•−^, in which O_3_ reacts with H_2_O to generate •OH, and O_3_ activates PMS to produce SO_4_^•−^. SO_4_^•−^ and •OH co-oxidize pollutants, which makes it possible to treat more complex organic pollution in water environment. SO_4_^•−^ and •OH have strong oxidizability, so the process has the advantages of rapidly degrading organic pollutants and mineralizing the same into CO_2_ and H_2_O. Meanwhile, for the research of the O_3_/PMS process, the generation of by-product BrO_3_^−^ should be considered. In the reaction, a low dosage of carbon material can reduce HOBr/OBr^−^ to Br^−^, thereby inhibiting the generation of bromate.

This paper focuses on the treatment efficiency of O_3_/PMS and its composite system for different kinds of antibiotic pollutants in drinking water and wastewater effluent. The addition of a catalyst composite can generate more •OH radicals and SO_4_^•−^ radicals. The addition of carbon materials can inhibit the generation of by-products, greatly shorten the reaction time, and improve the degradation efficiency. This also can realize the efficient removal of antibiotic pollutants in the water environment. This has great implications for protecting water resources and ecosystems and reducing human health risks.

## 4. Future Directions

The O_3_/PMS process has wide application potential in water treatment, and future directions for research include the following: Firstly, it is necessary to combine other technologies on the basis of the O_3_/PMS system, which can improve the degradation efficiency to some extent [60]. Secondly, researchers should look for or independently develop economic and environmental catalyst materials. By adding a catalyst composite and optimizing reaction conditions, the process can be cleaner and more efficient, and the catalytic activity and stability of the system can be further improved [61]. Thirdly, the evaluation of side reactions and toxicity of intermediate products in the process of degradation needs further study, including in the areas of degradation mechanisms and degradation pathways. It is necessary to explore more targeted treatment methods to degrade pollutants and reduce toxic by-products from the treatment of antibiotic wastewater [57]. At the same time, it is important to develop a combined process with other water treatment technologies [62], and to deeply study the scale of AOPs process to protect human health and ecological environment.

## Figures and Tables

**Table 1:** Comparison of mainstream advanced oxidation technologies.

Process	Advantages	Disadvantages	References
O_3_/PMS oxidation	Simple equipment, mild reaction conditions, and normal temperature and pressure operation; the production of two active free radicals, strong oxidation ability, high efficiency and stronger applicability.	Weak mineralization ability, and the running in weak alkaline environment.	[19]
Fenton oxidation	Friendly to the environment, occupies a small space, high oxidation efficiency and strong oxidation ability.	High treatment cost, harsh operating conditions and the running in strong acid conditions.	[20]
Photochemical catalytic oxidation	Green and environment-friendly lighting, little impact on water quality, low operating cost and long service life.	Needs to be combined with other technologies to achieve better results: such as UV/H_2_O_2_, and UV/PDS; ultraviolet light beam with built-in light tube harmful to human body.	[21]
Electrochemical oxidation	Simple electrolysis device, convenient operation and control, the oxidization of pollutants by the anode, and the changes of the anode material to destroy different types of organic matter.	Excessive energy consumption, low reactor efficiency and high equipment cost.	[22]
O_3_/H_2_O_2_ oxidation	Fast oxidation speed and high oxidation efficiency.	Complex equipment, high energy consumption, high cost and easy to produce bromate.	[23]

**Table 2:** Reaction equation for the generation of •OH in O_3_/H_2_O_2_ system.

Equation	Serial Number
$H{O_{2}}^{-}+O_{3}\to H{O_{5}}^{-}$	(1)
$H{O_{5}}^{-}\to {O_{3}}^{•-}+H{O_{2}}^{•}$	(2)
$H{O_{5}}^{-}\to 2O_{2}+OH^{-}$	(3)
${O_{3}}^{•-}⇌O^{•-}+O_{2}$	(4)
$O^{•-}+H_{2}O⇌•OH+OH^{-}$	(5)
$H{O_{2}}^{•}⇌{O_{2}}^{•-}+H^{+}$	(6)
${O_{2}}^{•-}+O_{3}\to {O_{3}}^{•-}+O_{2}$	(7)

**Table 3:** Reaction equation and reaction rate constant for the generation of •OH and SO_4_^•−^ in the O_3_/PMS system.

Equation	Reaction Rate Constant (L·mol^−1^·s^−1^)	Serial Number
$-O_{3}S{OO}^{-}+O_{3}\to -O_{3}S{O_{5}}^{-}$	$2.12\times 10^{4}$	(8)
$-O_{3}S{O_{5}}^{-}\to S{O_{5}}^{•-}+{O_{3}}^{•-}$	none	(9)
$-O_{3}S{O_{5}}^{-}\to S{O_{4}}^{2-}+2O_{2}$	none	(10)
$S{O_{5}}^{•-}+O_{3}\to S{O_{4}}^{•-}+2O_{2}$	$1.6\times 10^{5}$	(11)
$2S{O_{5}}^{•-}\to 2S{O_{4}}^{•-}+O_{2}$	$2.1\times 10^{8}$	(12)
$2S{O_{5}}^{•-}\to S_{2}{O_{8}}^{2-}+O_{2}$	$2.1\times 10^{8}$	(13)
${O_{3}}^{•-}⇌O^{•-}+O_{2}$	none	(14)
$O^{•-}+H_{2}O\to •OH+OH^{-}$	none	(15)
$HS{O_{5}}^{-}+H_{2}O\to H_{2}O_{2}+HS{O_{4}}^{-}$	none	(16)
$S{O_{5}}^{2-}+H_{2}O\to H_{2}O_{2}+S{O_{4}}^{2-}$	none	(17)
$2O_{3}+H_{2}O_{2}\to 2•OH+3O_{2}$	none	(18)
$S{O_{4}}^{•-}+OH^{-}\to S{O_{4}}^{2-}+•OH$	$(6.5\pm 1.0)\times 10^{7}$	(19)
$S{O_{4}}^{•-}+H_{2}O\to H^{+}+S{O_{4}}^{-}+•OH$	$<3\times 10^{3}$	(20)
$S{O_{5}}^{2-}+HS{O_{5}}^{-}\to HS{O_{4}}^{-}+S{O_{4}}^{2-}+O_{2}$	none	(21)

## Data Availability

Data are contained within the article.
